# Serologic and Molecular Evidence of Vaccinia Virus Circulation among Small Mammals from Different Biomes, Brazil

**DOI:** 10.3201/eid2306.161643

**Published:** 2017-06

**Authors:** Júlia B. Miranda, Iara A. Borges, Samantha P.S. Campos, Flávia N. Vieira, Tatiana M.F. de Ázara, Fernanda A. Marques, Galileu B. Costa, Ana Paula M.F. Luis, Jaqueline S. de Oliveira, Paulo César P. Ferreira, Cláudio Antônio Bonjardim, Silvio L.M. da Silva, Álvaro E. Eiras, Jônatas S. Abrahão, Erna G. Kroon, Betânia P. Drumond, Adriano P. Paglia, Giliane de S. Trindade

**Affiliations:** Universidade Federal de Minas Gerais, Belo Horizonte, Brazil (J.B. Miranda, I.A. Borges, S.P.S. Campos, F.N. Vieira, T.M.F de Ázara, F.A. Marques, G.B. Costa, A.P.M.F. Luis, J.S. de Oliveira, P.C.P. Ferreira, C.A. Bonjardim, A.E. Eiras, J.S. Abrahão, E.G. Kroon, B.P. Drumond, A.P. Paglia, G.D.S. Trindade);; Instituto Federal de Educação, Ciência e Tecnologia do Sudeste de Minas Gerais, Rio Pomba, Brazil (S.L.M. da Silva)

**Keywords:** vaccinia virus, VACV, orthopoxvirus, bovine vaccinia, rodents, marsupials, hosts, viral ecology, viruses, Brazil

## Abstract

Vaccinia virus (VACV) is a zoonotic agent that causes a disease called bovine vaccinia, which is detected mainly in milking cattle and humans in close contact with these animals. Even though many aspects of VACV infection have been described, much is still unknown about its circulation in the environment and its natural hosts/reservoirs. To investigate the presence of *Orthopoxvirus* antibodies or VACV DNA, we captured small rodents and marsupials in 3 areas of Minas Gerais state, Brazil, and tested their samples in a laboratory. A total of 336 animals were tested; positivity ranged from 18.1% to 25.5% in the 3 studied regions located in different biomes, including the Atlantic Forest and the Cerrado. Analysis of nucleotide sequences indicated co-circulation of VACV groups I and II. Our findings reinforce the possible role played by rodents and marsupials in VACV maintenance and its transmission chain.

Virus species belonging to genus *Orthopoxvirus* (OPV) receive great attention because of *Variola virus* (VARV), which is associated with smallpox (*1*). Smallpox caused many deaths worldwide and was eradicated after a massive vaccination campaign developed by the World Health Organization (*2*). Because OPVs have very similar antigenic structure (*1*), cross-protection enabled the use of cowpox virus (CPXV) and later vaccinia virus (VACV) as anti-smallpox vaccine agents (*2*). 

Given its widespread use, VACV has been studied for many years, and these efforts shed light on various aspects regarding virus biology. After smallpox eradication, vaccination was discontinued (*2*), and only select institutions in the United States (e.g., the military and certain public health facilities) receive the vaccine for their efforts to prevent the use of VARV as a biologic weapon. Household transmission from vaccinees and eczema vaccinatum are some of the negative aspects of vaccinating and have been responsible for severe outcomes (*3*). Although VARV is now restricted to laboratory facilities, other OPVs have been emerging as zoonotic pathogens in different geographic areas, namely CPXV in Europe, monkeypox virus (MPXV) in Africa, and VACV in Asia and South America (*4*). 

In Brazil, natural infections with VACV are called bovine vaccinia (BV) and are reported in rural areas, mainly in milking cattle and in men who are in close contact with these animals. The first officially recorded reports of BV date from the early 2000s and occurred in the southeastern region of Brazil (*5*,*6*). Currently, there is evidence of virus circulation in all regions of Brazil (*7*,*8*); however, the southeast is still the epicenter of registered BV cases, with Minas Gerais state being one of the most affected. Studies have shown that mammal species in addition to bovids and humans could be naturally infected by (or at least exposed to) VACV (*9*–*19*). VACV was isolated from samples from a rodent from the Amazon region in the 1960s (*9*), and now there are other documented incidents of virus circulation in these animals (*10*–*12*,*15*,*17*). By taking into account virus detection in small rodents, the fact that CPXV (*20*) and probably MPXV have rodents as reservoirs (*21*), and the frequent reports of these animals’ presence during BV outbreaks, an ecologic model was created to propose the participation of rodents in the VACV transmission chain (*12*). Because some species of native rodents could have ecologic advantages in areas with anthropic disturbance, they could work as bridges between natural and human/domestic habitats, bringing viruses from wild animals to domestic ones and vice versa (*12*). This model is reinforced by studies of virus transmission between mice and from experimental infection through contaminated milk (*22*). To better evaluate the circulation of VACV in small rodents, we undertook comprehensive collection campaigns in 3 areas of Minas Gerais with or without confirmed BV outbreaks. Animals were evaluated for the presence of VACV DNA and antibodies against OPV. Because marsupials were often captured and previous studies have detected OPV antibodies and VACV DNA in these animals (*17*,*19*), their samples were also tested.

## Methods

### Collection Sites

**Figure 1:**
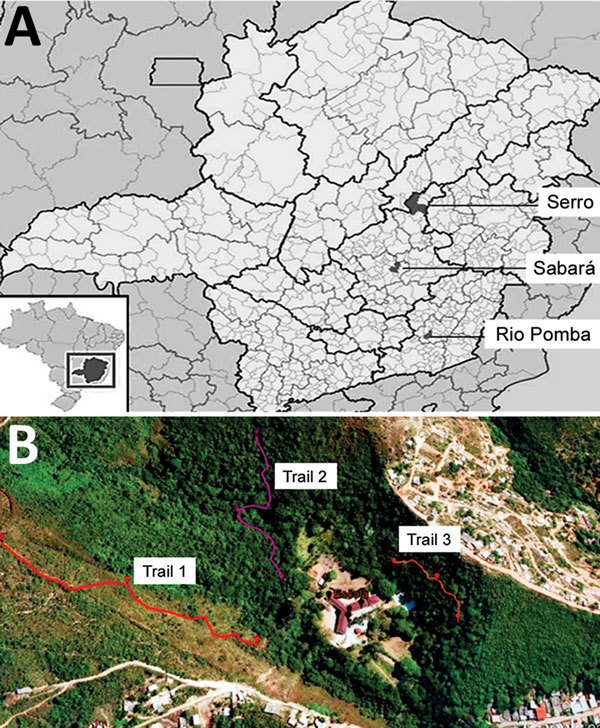
Locations of study areas, Minas Gerais state, Brazil. A) Locations of the 3 municipalities where collections were performed: Sabará, Serro, and Rio Pomba. Inset shows location of Minas Gerais state in southeastern Brazil. B) Identification of 3 sample transects in Sabará. Trail 1 has savannah vegetation, and trails 2 and 3 have Atlantic Forest vegetation. Sources: panel A, Scribble Maps; panel B, T.M.F. de Ázara.

We collected small mammals in 3 areas of Minas Gerais. Brazil was chosen because of its history of BV outbreaks and its different biomes and conservational status (Figure 1, panel A). The 3 municipalities where collections were performed were Sabará, Serro, and Rio Pomba. 

Sabará is a city located in an anthropic area situated in the transition from savannah (the Cerrado biome) to the Atlantic Forest. The study site (19°53′9′′S, 43°48′45′′W) was delimited on the grounds of a former educational institution in a previous study (Figure 1, panel B). Three sampling transects were demarcated: 1 in savannah vegetation with intense anthropogenic disturbance and the other 2 in forest vegetation (with 1 of the 2 having more disturbance than the other) (Figure 1, panel B). In each transect, 15 sampling points were established with 2 live traps in each, 20 m apart, where captures of small mammal took place. 

**Figure 2:**
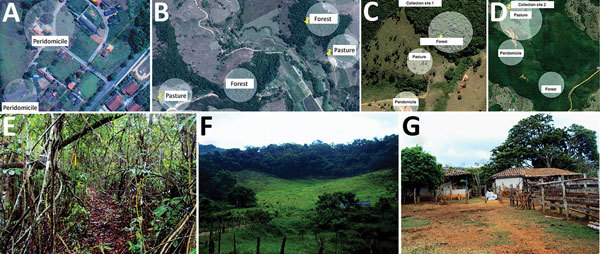
Location of collection sites and biomes represented in each, Minas Gerais state, Brazil. A) Collection site 1 in Serro. B) Collection site 2 in Serro. C) Peridomicile collection areas in Rio Pomba. D) Forest and pasture collection areas in Rio Pomba. E) Example of a forest area where animals were captured. F) Example of peridomicile area. G) Example of pasture area. In panels A–D, circles represent areas where transects for capture were demarcated. Sources: panels A,–D, Google Maps, modified by F.V. Nunes; panels E–G, F.V. Nunes.

In Rio Pomba, the field site (21°16′29′′S, 43°10′45′′W) has characteristic Atlantic Forest vegetation. Animal trapping was performed in the area around the Instituto Federal de Educação, Ciência e Tecnologia (Figure 2, panels A, B). Animal collections were performed in forest, pasture, and peridomicile areas (Figure 2, panels A–G). In each transect, 10 traps were placed at a distance of 10 m from each other and in alternating positions (on forest floor or on tree trunks). 

Serro, a city whose economy is based on milk and cheese production, has seen many cases of BV since 2005 (*23*). The capture of small mammals was carried out in 2 farms (Figure 2, panels C, D). The study site (180°36′21.16′′S, 43°23′12.89′′W) is situated in the Cerrado biome and has some intersections of Atlantic Forest. Animal collections were carried out in forest, pasture, and peridomicile areas (Figure 2, panels A–G), and traps were positioned in the same manner as in Rio Pomba.

### Animal Trap and Sample Collection

Captures lasted from April 2011 through May 2012 for Sabará (12 campaigns) and from September 2012 through September 2013 in Serro (5 campaigns) and Rio Pomba (6 campaigns). Small mammals were captured in size-selective live cages by using pineapple chunks and cotton balls soaked in cod liver oil as baits. Each sampling section lasted for 4 nights, and baits were replaced after 2 nights. After capture, animals were anesthetized with ketamine (70 mg/kg) and xylazine (12 mg/kg) for serum collection. Animals were weighed, measured for size, and visually evaluated for clinical signs of disease, such as skin lesions. For organ collection, animals were euthanized by intracardiac injection of 3 times the anesthetic dose according to guidelines of the American Society of Mammalogists (*24*). Collections were authorized by the Environment Ministry of Brazil through the SISBIO system (license no. 20807–2).

### Biosafety

All collections were performed by trained professionals (either veterinarians or biologists) according to US Centers for Disease Control and Prevention recommendations (*25*). During animal manipulation, personal protective equipment (disposable coveralls, surgical gloves, goggles, and N98 masks) was used.

### DNA Extraction from Organs

In addition to serum, which was tested by real-time PCR (rPCR) targeting the C11R viral growth factor gene without DNA extraction, liver was the chosen organ for rPCR trials. The organs were macerated with mortar and pestle after liquid nitrogen was added, and DNA was extracted with PureLink Genomic DNA Mini Kit (Invitrogen, Carlsbad, CA, USA) as recommended by the manufacturer. The same protocol was applied for other organs tested, including intestine, bladder, heart, gonads (ovary/testicles), bone marrow, spleen, lung, diaphragm, and kidney.

### Cells and Virus

A VACV Western Reserve strain was used as a positive control. BSC-40 cells were grown in Eagle’s minimum essential medium (Invitrogen) supplemented with 5% fetal bovine serum (Cultilab, São Paulo, Brazil); 25 mg/mL Fungizone (amphotericin B) (Cristália, São Paulo, Brazil); 500 U/mL penicillin; and 50 mg/mL gentamicin (Schering-Plough, São Paulo, Brazil).

### rPCR Assays

All rPCR experiments were performed in 48-well plates in Step One machines (Applied Biosystems, Foster City, CA, USA) by using SYBR Green Master Mix (Applied Biosystems). DNA from liver samples was diluted in water for a final use concentration of 10 ng/μL and 50 ng/μL. For serum samples, a 1:10 or 1:100 dilution was performed, and samples were tested without previous DNA extraction. For both liver and serum samples, amplification of the C11R gene was tested, and liver samples were additionally tested for amplification of the A56R hemagglutinin gene (primer sequences available upon request). For C11R, an amplicon of 82 bp and a melting temperature of 74.99°C were expected, and for A56R, a sequence of 160 bp and a melting temperature of 74.41°C were expected. All reactions had a final volume of 10 μL, and samples were tested in duplicates. Reaction steps comprised initial DNA denaturation at 95°C for 10 min, 40 cycles of denaturation (95°C for 15 s), annealing/extension (60°C for 60 s), and a melting curve (95°C for 15 s, 60°C for 60 s, and 95°C for 15 s). Samples were considered positive when melting temperature varied only up to 1°C compared with a positive control (10 ng of DNA extracted from purified VACV Western Reserve strain) and had amplification in duplicate or for >1 target. Samples with a single amplification were retested and considered equivocal when no more amplification was observed.

### Nucleotide Sequencing and Sequence Analyses

Positive samples that could be reamplified (A56R-positive) or amplified by a conventional PCR targeting C11R (C11R-positive) (*26*) were chosen for sequencing. For A56R, product from the previous reaction was reamplified in a conventional PCR reaction using 1 μL of the first reaction as input and 0.2 nmol/L (A56R) rPCR primers. PCR cycling for A56R gene consisted of 10 min at 95°C for denaturation, 30 cycles of denaturation (95°C for 10 min), annealing (60°C for 60 s), extension (72°C for 60 s), and a final extension of 10 min at 72°C. Products with single bands were directly sequenced, and products with multiple bands had the target gene extracted from acrylamide gels stained with SYBR Gold Nucleic Acid Gel Stain (Invitrogen) and had its DNA purified. Nucleotide sequencing was performed by dideoxy method in an ABI3130 platform (Applied Biosystems), and sequence quality was analyzed by using Sequence Scanner Software 1.0 (Applied Biosystems). Sequences were aligned with other reference sequences from the BLAST nucleotide database (http://blast.ncbi.nlm.nih.gov/Blast.cgi) by using MEGA 6.0; the same program was used for identity matrix construction (*27*).

### Plaque-Reduction Neutralization Test 

The plaque-reduction neutralization test (PRNT) protocol has been described previously by Geessien Kroon et al. (*28*). Samples were considered positive when a reduction of >50% in virus plaque numbers was observed.

### ELISA

ELISA was performed for rodent blood samples following a protocol also described previously (*28*). For each plate, 1 positive control (serum from *Mus musculus* experimentally infected with VACV-Guarani P1) (*29*) and 3 negative controls (serum of noninfected *M. musculus*) were added. Cutoff was established as the mean of negative controls optical density units plus 3 times their SD. Samples with an optical density 10% above or below the cutoff were considered equivocal.

### Interaction Networks

Interaction networks are useful to help with understanding of virus-host dynamics. Each species is represented by a vertex, and the link between 2 vertices represents the interaction between 2 different species, making it possible to analyze their interdependence (*30*). The networks also show which species have the higher number of positive samples for virus detection and the area where they were collected. By using data from VACV-positive small mammal species, we created weighted networks with the program Pajek 4.07 (*31*). Accordingly, adjacency matrices were generated for each study area in which hosts were represented by lines and VACV by columns.

## Results

### rPCR Amplification of VACV DNA from Free-living Small Mammals

A total of 325 animals had their samples tested by rPCR targeting the C11R gene, the A56R gene, or both. Of these animals, 21 (6.4%) tested positive (i.e., amplification in duplicates or in >1 sample/target) and 58 (17.8%) equivocal (Technical Appendix Table 1). The cycle thresholds varied from 28.42 to 39.33. From the total animals tested by rPCR, 114 had samples available for all tests (C11R targeted in liver and serum and A56R in liver). One animal was positive in the 3 tests performed, 11 in 2 tests, and 5 in only 1 test (data not shown). For the remaining positive animals, >1 assays could not be performed, and 1 sample type was positive in 1 test. 

Of all the animals from the different collection sites in Sabará, 11/48 (22.9%) rodents and 3/76 (3.9%) marsupials were positive by rPCR. For Serro, no marsupials were positive but 4/25 (16.0%) rodents were positive. For Rio Pomba, 2/137 (1.4%) of rodents and 1/18 (5.5%) of marsupials were positive (Technical Appendix Table 1). 

Four rPCR-positive animals (2 rodents and 2 marsupials) were selected for viral DNA detection in different organs by rPCR targeting the C11R and A56R genes. Positivity was found for heart, spleen, intestines, bladder, lungs, kidneys, and gonads (Technical Appendix Table 2). No amplification was observed in any bone marrow or diaphragm samples tested.

### OPV Antibodies in Serum from Free-living Small Mammals Tested by PRNT

PRNT tests were performed in a total of 314 serum samples, and from these, 33 were considered positive, corresponding to 10.5% of the animals. The reduction percentages varied from 50.5% to 95.6%. For the Sabará collection, positivity was 9.0% (10/111), 14.3% (6/42) for rodents and 5.8% (4/69) for marsupials. For Serro, positivity was 4.2% (2/47), and only rodent samples were positive, corresponding to 8.0% (2/25) of rodents tested. For Rio Pomba, positivity was 13.4% (21/156), 14.3% (20/139) for rodents and 5.8% (1/17) for marsupials (Technical Appendix Table 1).

### OPV Antibodies in Serum from Free-living Small Mammals Tested by ELISA

ELISA tests were performed on 189 rodent serum samples; a control serum for marsupials was not available. Of the animals tested, 19/189 (10.0%) were positive and 11/189 (5.8%) equivocal (Technical Appendix Table 1). By location, 3/35 (8.5%) animals from Sabará, 9/25 (36.0%) from Serro, and 7/129 (5.4%) from Rio Pomba were positive.

### Sequencing

Two PCR amplicons, amplified from Sabará animals, were sequenced with C11R primers and resulted in sequences of 168 bp that were aligned with VACVs in Brazil and other OPVs (Technical Appendix Figure 1). These 2 sequences had 100% similarity with each other; similarity with VACVs in Brazil ranged from 98.2% to 100% and with CPXV from 87.3% to 89.1%, whereas similarity with VARV was 94.5% and with MPXV 90.9% (data not shown). For A56R, sequencing was performed in positive rPCR samples and resulted in 6 sequences of 102 bp. When compared with OPV sequences, 2 samples from Sabará had an 18-nt deletion shared by Brazil VACV group I, whereas the other 4 samples from Sabará, Serro, and Rio Pomba did not have that deletion, being more similar to Brazil VACV group II and other OPVs (Technical Appendix Figure 2).

### Geographic and Species Distribution of Positivity

For all areas studied, 336 animals belonging to 18 genera had their samples tested by rPCR, PRNT, and/or ELISA, and 65 (19.3%) were positive in >1 of these tests. Total positivity was 18.1% for Sabará, 25.5% for Serro, and 18.5% for Rio Pomba (Technical Appendix Table 1). A higher positivity was observed for rodents (25.7%) than for marsupials (7.6%) (data not shown). 

Species identified among the test-positive rodents were *Calomys* sp., *Akodon* sp., *Necromys lasiurus*, *Trinomys setosus*, *Cerradomys subflavus*, *Oligoryzomys* sp., *Nectomys squamipes*, *Mus musculus*, and *Rattus rattus*. For marsupials, the positive animals were characterized as species/genera *Didelphis* sp., and *Caluromys philander* (Technical Appendix Table 3). 

**Figure 3:**
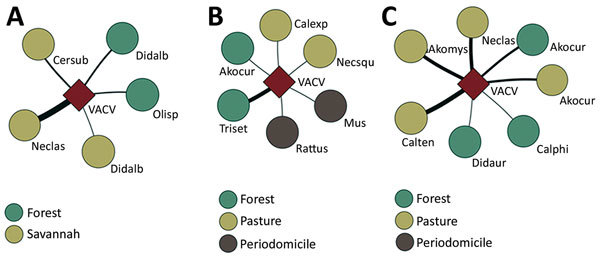
Interaction networks for vaccina virus among small mammals in Sabará (A), Serro (B), and Rio Pomba (C) in Minas Gerais state, Brazil. The square represents vaccinia virus. Circles represent small mammal species (labeled). The color in the circles represents the area where mammals were collected. The thickness of lines increases with the number of positive samples from a species. Acokur, *Akodon cursor* mouse; Akomys, *Akodon* cf; mystax; Calexp, *Calomys expulsus*; Calphi, *Caluromys philander*; Calten, *Calomys tener*; Cersub, *Cerradomys subflavus*; Didalb, *Didelphis albiventris*; Didaur, *Didelphis aurita*; Mus, *Mus musculus*; Neclas, *Necromys lasiurus*; Necsqui, *Nectomys squamipes*; Olisp, *Oligoryzomys* sp.; Rattus, *Rattus rattus*; Triset, *Trinomys setosus*; VACV, vaccinia virus.

Test-positive animals were captured in all sample areas in Sabará (savannah and forest) and Serro (pasture, forest, and peridomicile areas), whereas test-positive animals in Rio Pomba were captured in pasture and forest. The interaction network for Sabará illustrates that 4 species had positive samples, with 3 of them found either in forest or savannah and the other in both areas. *N. lasiurus* (the hairy-tailed bolo mouse) had the highest number of positive samples in Sabará (Figure 3, panel A). For Serro, evidence of VACV circulation was found in 6 species; of these, 2 were captured in forest, 2 in pasture, and 2 in peridomicile areas. The species with the largest number of positive samples in Serro was *T. setosus* (the hairy Atlantic spiny rat; Figure 3, panel B). For Rio Pomba, 5 genera were positive, including *Akodon* sp. mice captured in pasture and forest, *Calomys tener* and *N.* *lasiurus* mice captured in forest, and *C.* *philander* and *Didelphis* *aurita* opossums captured in pasture. The species with the highest number of positive animals in Rio Pomba was *C. tener* (the delicate vesper mouse), followed by *N. lasiurus* and *Akodon* sp. mice (Figure 3, panel C).

## Discussion

In our study, we analyzed different biomes in an area where BV infections are common, and the positivity rates found for VACV were 25.7% for rodents and 7.6% for marsupials. Even though VACV is known to circulate in Brazil and cause a disease that leads to economic, social, and public health effects, few studies have been conducted with the aim of clarifying the VACV transmission chain and potential natural hosts (*9*–*19*,*22*,*32*). Previous studies showed antibody positivity of 8.7%–17.9% for wild rodents captured in places with or without documented BV (*15*,*17*) and seropositivity of 8.2% for *Didelphis* spp. marsupials (*17*). 

The higher positivity rate found for rodents in our study could be attributable to the use of 3 different techniques, including 2 techniques for detecting antibodies (PRNT and ELISA) and 2 targets for DNA detection (rPCR).The 3 techniques used to assess virus circulation provide distinct responses about infection stages. Whereas rPCR indicates the presence of viral DNA, the ELISA used in our study reveals the presence of IgG (indicative of previous infection) (*33*), and PRNT detects neutralizing antibodies that can be of different types, including IgG and IgM, which are produced early in the infection process (*34*). Because we found positive animals for >1 techniques, we can speculate that an active transmission cycle is happening in all 3 study areas. Additionally, only Serro has recurrent reports of BV outbreaks (*23*,*29*), which could be a result of the presence of positive animals in the peridomicile area, where they could infect other animals, such as cows, and cause disease. Furthermore, because large-scale milk production occurs in Serro, many livestock animals, including bovines, could work as infection amplifiers.

The rPCR technique has been used for MPXV detection in rodents in Africa, where samples were considered equivocal when repeatability of results was not achieved (*21*). In our study, we made this same observation, which might be attributable to a low virus load in the samples. In turn, the low virus load could be related to the late cycle threshold in which amplification occurred and a lack of clinical signs in animals with a positive result.

Sequencing of A56R rPCR amplicons revealed the co-circulation of Brazil VACVs belonging to groups I and II, a fact that reinforces previous data on Brazil VACV virus diversity (*11*,*35*–*37*). Again, even when infected with virus belonging to group II, which were found to be virulent in a mice model (*38*), wild rodents and marsupials tested in our study did not have clinical signs detected. Also, these animals infected with VACV group I or II had viral DNA in many organs, as indicated by rPCR. It is not possible to assert that virus is replicating in these tissues, given that virus could be present in blood that circulates through these organs; however, previous in vivo infection experiments have found virus in different mice organs (*32*), probably because of systemic infection. This observation also was made in mice infected with milk (a possible route of natural infection) contaminated with VACV-Guarani P2 virus. This virus was found to be nonvirulent in a mice model; animals shed viral DNA and produced OPV antibodies but did not show clinical signs (*22*). The detection of virus DNA in intestines, bladder, and gonads could reinforce previous data suggesting that virus transmission occurs through feces (*39*) and support the hypothesis of alternative transmission through urine and sexual contact. Mariana virus has been isolated from the gonads of mice (*14*), so it could be speculated that the sexual transmission route is involved.

Among the positive rodent species, *Akodon* sp., *N. squamipes*, *Oligoryzomys* sp. (*15*,*17*), and *M. musculus* (*12*) have already been found to be positive in previous studies, reinforcing evidence of its participation in the VACV transmission cycle; however, the exact role played by these animals is not yet known. In addition to *M. musculus*, *C. subflavus*, *N.* *lasiurus*, *T.* *setosus*, *C.* *tener*, and *R.* *rattus* rodents were also found to be positive in our study, indicating the role of multiple hosts in VACV transmission in Brazil. Among the marsupials, *Didelphis* spp. opossums had already been found to be positive (*17*,*19*), and *C.* *philander* opossums also had positive samples.

Although only 1 virus (VACV) was analyzed for interaction network construction and no interactions between different species were observed, the networks created illustrate the participation of the small mammals for each studied area and the areas where these positive animals were collected (Figure 3). The networks also suggest an important role of *N.* *lasiurus* mice for the VACV transmission chain in Sabará, *T.* *setosus* rats in Serro, and *C.* *tener*, *N.* *lasiurus*, and *Akodon* sp. mice in Rio Pomba. Most of these species are generalist animals that can be adapted to a disturbed environment. However, the *C.* *philander* opossum is an arboreal species that lives in forests (*40*), which could indicate that a wild cycle is being maintained and that other animals could be transporting the virus between forests and peridomicile areas. These findings corroborate the models proposed by Abrahão et al. (*12*) in which rodents and other small mammals could work as links between natural and anthropic environments.

In conclusion, our findings reinforce evidence of participation of rodents and marsupials in the VACV transmission cycle and the possibility that these animals might work as links between natural and anthropic environments. These findings also further illustrate the multi-host characteristic of VACV infection in Brazil.

Technical Appendix Results of real-time PCR and serologic tests, comparison of vaccinia virus positivity rates among rodents and marsupials, and sequence alignment of C11R and A56R genes in samples from Sabará, Serro, and Rio Pomba.
