# Supplementary material for: Serologic and Molecular Evidence of Vaccinia Virus Circulation among Small Mammals from Different Biomes, Brazil
Source: Emerg Infect Dis. 2017 Jun;23(6):931–8. doi: 10.3201/eid2306.161643 (PMC5443434; doi:10.3201/eid2306.161643)
Supplement: Technical Appendix — Results of real-time PCR and serologic tests, comparison of vaccinia virus positivity rates among rodents and marsupials, and sequence alignment of C11R and A56R genes in samples from Sabará, Serro, and Rio Pomba. [file 16-1643-Techapp-s1.pdf]

Article DOI: https://dx.doi.org/10.3201/eid2306.161643

Serologic and Molecular Evidence of Vaccinia Virus Circulation among Small Mammals from Different Biomes, Brazil

Technical Appendix

Technical Appendix Table 1. Results of qPCR (C11R and A56R genes) and serologic (PRNT and ELISA) tests for each collection site

Collection area/ Morphoclimatic domain	Animals	qPCR			PRNT		ELISA			Combined results	
		Tested [L/S]	Equivocal [L/S] (%)	Positive [L/S] (%)	Tested	Positive (%)	Tested	Equivocal (%)	Positive (%)	Tested	Positive (%)
Sabará (SA)/ Transition Savannah/ Atlantic forest	Rodents	48 [35/46]	21 [18/3] (43.7)	11 [10/7] (22.9)	42	6 (14.3)	35	5 (14.3)	3 (8.5)	49	16 (32.6)
	Marsupials	76 [5/74]	7 [4/3] (9.2)	3 [0/3] (3.9)	69	4 (5.8)	NT	NT	NT	78	7 (8.9)
	Total	124 [40/120]	28 [22/6] (22.6)	14 [10/10] (11.3)	111	10 (9.0)	–	–	–	127	23 (18.1)
Serro (SE)/ Savannah	Rodents	25 [20/25]	4 [1/3] (16.0)	4 [3/3] (16.0)	25	2 (8.0)	25	2 (8.0)	9 (36.0)	25	12 (48.0)
	Marsupials	21 [1/21]	1 [1/0] (4.7)	0	22	0	NT	NT	NT	22	0
	Total	46 [21/46]	5 [2/3] (10.8)	4 [3/3] (8.7)	47	2 (4.2)	–	–	–	47	12 (25.5)
Rio Pomba (RP)/ Atlantic forest	Rodents	137 [65/132]	24 [6/18] (17.5)	2 [2/0] (1.4)	139	20 (14.3)	129	4 (3.1)	7 (5.4)	144	28 (19.4)
	Marsupials	18 [2/18]	1 [0/1] (12.5)	1 [1/1] (5.5)	17	1 (5.8)	NT	NT	NT	18	2 (11.1)
	Total	155 [67/150]	25 [6/19] (16.1)	3 [3/1] (1.9)	156	21 (13.4)	–	–	–	162	30 (18.5)
Total		325 [128/316]	58 [30/28] (17.8)	21 [16/14] (6.4)	314	33 (10.5)	189	11 (5.8)	19 (10.0)	336	65 (19.3)

Technical Appendix Table 2. Results for qPCR amplification targeting C11R and A56R genes in distinct organs from four positive animals.

	S3 - *Didelphis albiventris* (female)		S211 - *Necromys lasiurus* (male)		SE151 - *Trinomys setosus* (female)		RP139 - *Caluromys philander* (male)	
Organ tested	C11R	A56R	C11R	A56R	C11R	A56R	C11R	A56R
Heart	–	–	+	+	+	–	+	–
Spleen	–	–	+	–	+	+	+	–
Intestin	+	–	+	+	+	–	+	–
Bladder	+	–	–	–	–	–	+	–
Lung	–	–	–	–	–	–	+	–
Kidney	+	–	NT	NT	+	–	+	NT
Gonad	+	–	–	–	+	+	+	+
Diaphragm	–	–	NT	NT	NT	NT	NT	NT
Bone marrow	NT	NT	–	–	NT	NT	NT	NT

+: Positive. -: Negative. NT: not tested because sample was not available. S: Sabará. SE: Serro. RP: Rio Pomba.

Technical Appendix Table 3. Positivity among rodents and marsupials for molecular and serologic tests

Animal	Species/genera	Collection areas	qPCR P/T (%)	PRNT P/T (%)	ELISA P/T (%)	Grouped results P/T (%)
Rodents	*Calomys* sp.	SE, RP	2 /71 (2.8)	8 /73 (10.9)	0 /69	9/77 (11.7)
	Akodon sp.	SE, RP	0 /46	10 /46 (21.7)	6 /43 (13.9)	15/46 (32.6)
	Necromys lasiurus	SA, RP	9 /45 (20.0)	9 /42 (21.4)	3 /33 (9.0)	17/47 (36.1)
	Trinomys setosus	SE	2 /8 (25.0)	0 /8	7 /8 (87.5)	7/8 (87.5)
	Cerradomys subflavus	SA, SE	3 /12 (25.0)	0 /12	0 /11	3/12 (25)
	Oligoryzomys sp.	SA, SE, RP	0 /14	1 /11 (9.0)	1 /13 (7.7)	2/14 (14.3)
	Nectomys squamipes	SE, RP	0 /2	0 /2	1 /2 (50)	1/2 (50)
	Mus musculus	SE	1 /3 (33.3)	0 /3	0 /3	1/3 (33.3)
	Rattus	SE	0 /3	0 /3	1 /3 (33.3)	1/3 (33.3)
	Guerlinguetus sp.	SA, RP	0 /2	0 /2	0 /0	0/2
	Oecomys catherinae	SE	0 /1	0 /1	0 /1	0/1
	Juliomys pictipes	RP	0 /2	0 /2	0 /2	0/2
	Oxymycterus sp.	RP	0/1	0/1	0/1	0/1
Marsupials	*Didelphis* sp.	SA, SE, RP	3 /90 (3.3)	5 /88 (5.6)	NT	8/93 (8.6)
	Caluromys philander	SE, RP	1 /2 (50)	0/2	NT	1/2 (50)
	Marmosops incanus	SE	0 /6	0 /6	NT	0/6
	Gracilinanus sp.	SA, SE, RP	0 /15	0 /10	NT	0/15
	Monodelphis americana	RP	0 /1	0/1	NT	0/1

Positivity found for each species/genera for qPCR, PRNT and ELISA, showing the grouped results for all the tests and the collection areas where the positive animals were captured. P/T: Positive/Tested. SA: Sabará. SE: Serro. RP: Rio Pomba. qPCR: Real time PCR. PRNT: Plaque reduction neutralizing test. ELISA: Enzyme linked immunosorbent assay. NT: Not tested *ELISA test was not performed for marsupial samples due a lack of control samples.

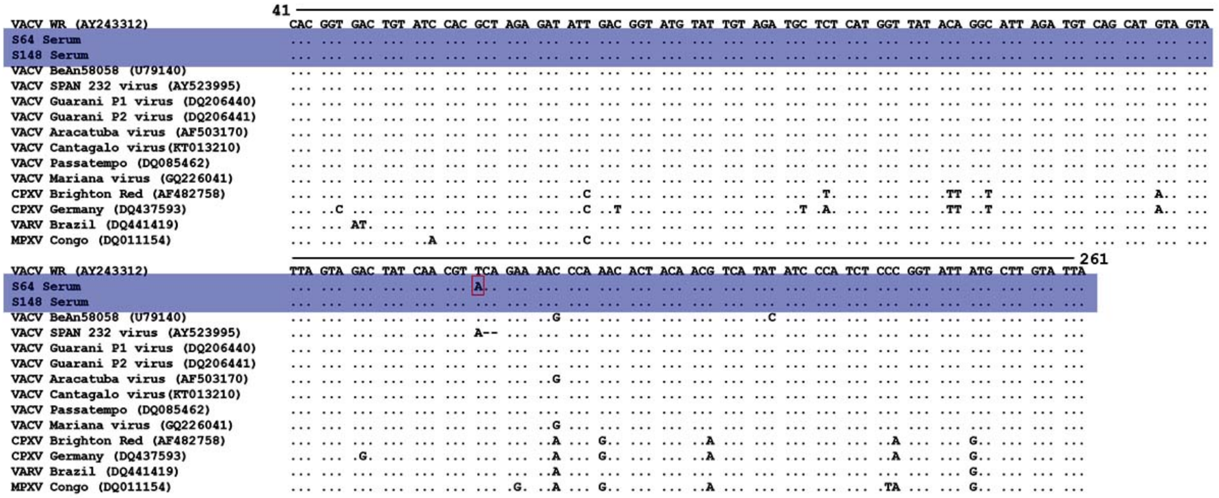

Technical Appendix Figure 1. Sequence alignment of gene C11R (vgf) for two Sabará serum samples (represented in blue) with Brazilian VACVs and other OPVs. Gene position is represented above alignments. The red square evidences a SNP from a Sabará sample that is shared by VACV SPAn 232 virus. Dots represent conserved bases, dash deletions and letters the divergent bases. S64 and S148 correspond to animals of the species *Didelphis albiventris*. VACV: Vaccinia virus. CXPV: Cowpox virus. VARV: Variola virus. MPXV: Monkeypox virus.

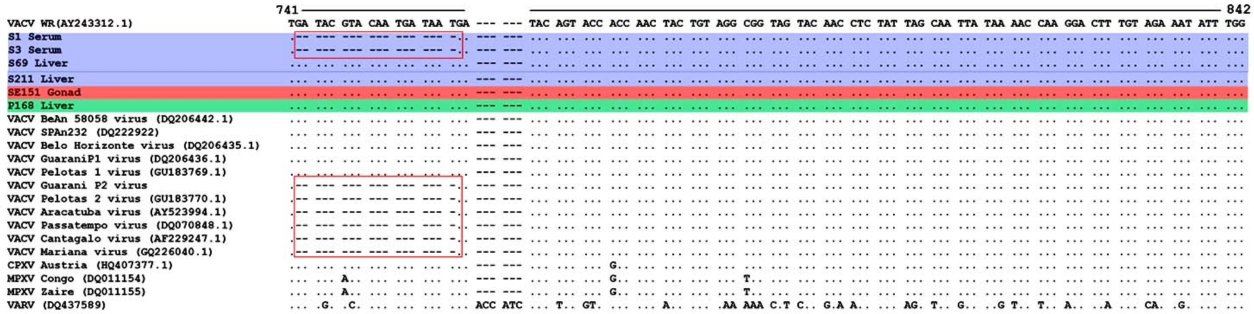

Technical Appendix Figure 2. Sequence alignment of A56R (ha) gene of samples from Sabará (blue), Serro (red) and Rio Pomba (green) and sequences of Brazilian VACVs and other OPVs. Conserved bases are represented by dots, divergent bases by letters and deletions by dashes. Red squares evidence the 18 nt deletion shared by group I of Brazilian VACVs and two samples from Sabará. Gene position is represented above alignments. Sequences from Sabará samples are shown as S, Serro as SE and Rio Pomba as RP. The species codified by S, SE and RP are: S1 *Cerradomys subflavus*, S3 *Didelphis albiventris*, S69 *Cerradomys subflavus*, S211 *Necromys lasiurus*, SE151 *Trinomys setosus* and RP 168 *Calomys tener*. VACV: Vaccinia virus. CXPV: Cowpox virus. VARV: Variola virus. MPXV: Monkeypox virus. Gonad of SE151 corresponds to ovary.
